# Objective Noninvasive Measurement of the Volumizing Effect of a Dermal Filler: An In Vivo Study

**DOI:** 10.1007/s00266-024-04138-3

**Published:** 2024-05-28

**Authors:** Xiaowen Liu, Huanyun Niu, Mengrou Shi, Bin Chen, Xin Li, Shiwei Wang, Jizhen Ren

**Affiliations:** 1 Department of Medical, Imeik Technology Development Co., Ltd., Beijing, China; 2 Tianjin Institute of Medical and Pharmaceutical Sciences, Tianjin, China; 3 The Department of Plastic and Cosmetic Surgery, The Affiliated Hospital of Qingdao University, No.16 Jiangsu Road, Qingdao, Shangdong Province China

**Keywords:** Dermal filler, Noninvasive measurement, Animal model

## Abstract

**Background:**

Information about the volumizing effects of dermal fillers is critical for physicians’ understanding of product features and prudent decision-making in clinical practice. It is important for material engineers to develop and optimize new dermal fillers, especially when comparing the physiochemical properties of a new product with those of existing fillers that are used worldwide.

**Objective:**

This study aimed to establish a reliable, noninvasive method for in vivo quantitative evaluation of the filling effect in order to predict possible effectiveness after filler injection and to evaluate the degradation trend over time.

**Methods:**

A rabbit model of ear injection with dermal fillers was established. Hyaluronic acid (HA) filler was injected into the subcutaneous layer of rabbit ears, resulting in a stable skin bulge. Ultrasonography was used to noninvasively measure the skin bulge for volume calculation; the volume change was analyzed periodically until 38 weeks. Pathological examination, the gold standard, was performed to confirm degradation.

**Results:**

The immediate volumizing effect of HA filler injection was macroscopically observed as a local skin bulge. Ultrasound was able to precisely detect the shape of the filler and calculate the length, width, and height of the skin bulge at each time point. The degree of uplift and amount of residual samples in the pathological evaluation were consistent with the results of morphological observation using ultrasound.

**Conclusion:**

Evaluation of the volume impact of dermal filler through the rabbit ear injection model evaluation enables material science evaluation in the early stage of material development, and has certain clinical reference value.

**Level of Evidence I:**

This journal requires that authors assign a level of evidence to each article. For a full description of these Evidence-Based Medicine ratings, please refer to the Table of Contents or the online Instructions to Authors www.springer.com/00266.

## Introduction

In the past decades, sodium hyaluronate-based fillers have been widely promoted because of their good biocompatibility and reversibility and are increasingly accepted by patients owing to the minimally invasive procedure and rapid recovery [1, 2]. However, the maintenance and effectiveness of filling effects remain a concern, and the filling effect may gradually weaken over time [3]. It is particularly important to evaluate the maintenance of the filling effect and preliminarily evaluate the filling validity period of the product during the preclinical stage [4, 5]. In the clinic, a variety of imaging techniques, such as high-frequency ultrasound and dermoscopy, have been used to assist in evaluating the effect of facial rejuvenation and diagnosing complications [6–8]. Ultrasound, a noninvasive soft tissue imaging technique, can detect a variety of echoes, obtain high-resolution images, and visualize skin and subcutaneous tissue changes due to different tissue densities [9, 10]. It has been widely used in the evaluation of the efficacy of injectable dermal fillers. Pasqual et al. [11] first used ultrasonography to evaluate the effects of medical cosmetic body fillers. Because HA fillers are hypoechoic under ultrasound, the volume and positional changes of the fillers can be measured using this method. Evaluation of the filling properties of materials, including the implantation effect of fillers, can provide guidance for material exploration and clinical use. For material engineers, it is more important to understand the test data of materials in vivo than in vitro. Compared with the in vitro measurement of the elastic modulus and other data, the supporting properties of fillers injected into animals can more conveniently and accurately reflect the chemical properties of the materials [12]. It is more suitable and convenient to establish an animal model than use ultrasonic detection to evaluate the filling effect after implantation in clinical patients. At present, preclinical animal experiments focus on the safety of products [13], and through pathological detection, the stimulatory effect and mechanism of products on skin hyperplasia are preliminarily explored [14, 15]. This study aimed to establish a reliable noninvasive measurement method for the in vivo quantitative evaluation of the filling effect in early product development, in order to evaluate the degradation trend over time to provide relevant data for clinical trial studies.

## Methods

### Materials

We used medical sodium hyaluronate–hydroxypropyl methylcellulose gel (EME Plus), which was developed by Imeik Technology Development Co., Ltd (Beijing, China) and approved by the Chinese National Medical Products Administration for correcting moderate to severe frontal wrinkles and nasolabial wrinkles.

### Animals

Fifteen male Japanese big-ear white rabbits (age, 4–5 months; weight, 2.2–2.5 kg) were provided by the Longan Laboratory Animal Breeding Center (Beijing, China). The rabbits had free access to food and water and were housed in metal cages with natural light, temperature of 20–25 °C, and 40–70% relative humidity. All applicable institutional and/or national guidelines for the care and use of animals were followed.

### Establishment of Animal Model

**Fig. 1:**
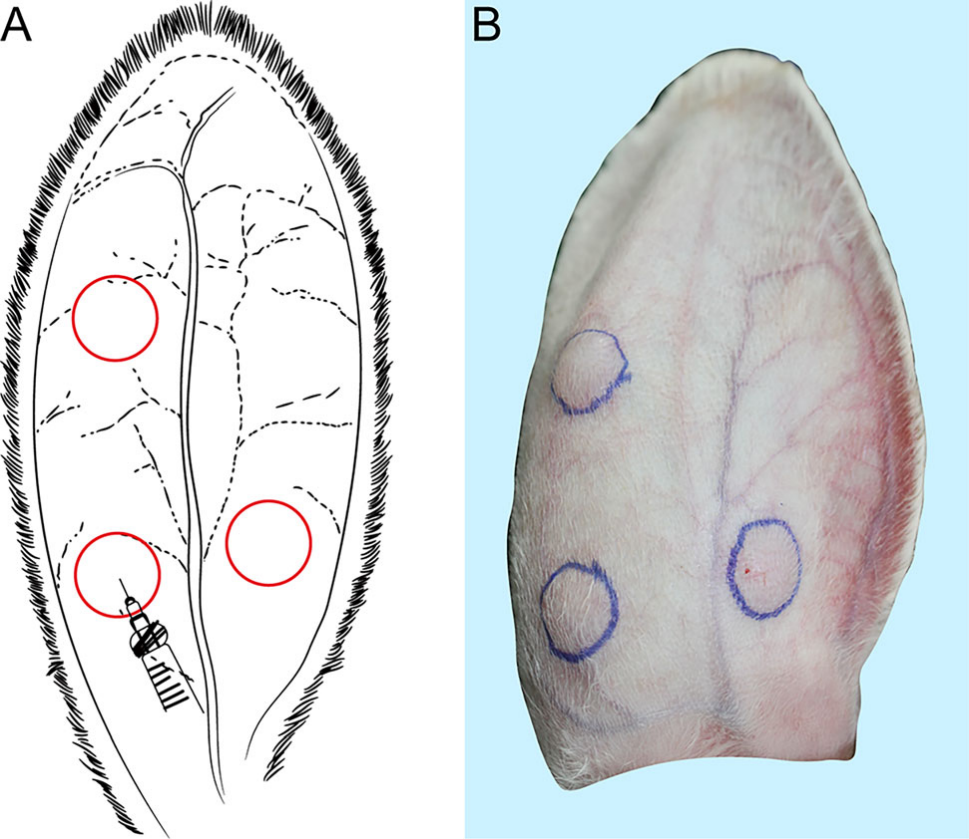
Rabbit ear injection. **A** Schematic model; **B** a corresponding injection picture of rabbit ear; three circles indicating injection sites. One out of the three sites that avoid blood vessels was selected and injected either medical hyaluronic acid sodium–hydroxypropyl methylcellulose gel

The epidermal layer thickness, elastic properties, and relaxation curve of rabbit skin are similar to those of human skin, implying that rabbit skin is a suitable candidate for preclinical dermatological trials [16]. In addition, the exceptionally thin auricular tissue of rabbit ears provides high observability for experimental purposes [17]. We selected the rabbit ear as the site of injection for this experiment, and the injected materials were administered between the epidermis and the ear cartilage using a 24-G needle. Specifically, 0.15 mL of medical sodium hyaluronate–hydroxypropyl methylcellulose gel was injected into the middle or proximal part of the dorsal ear dermis of the rabbits to form a bulge structure that could be accurately tracked in terms of location (Fig. 1). The gradual degradation of the material at the injection site and subsequent stimulation of tissue regeneration consequently lead to changes in the pico-structure and 3-dimensional shape of the implant.

### Measurements

The measurements were conducted by the same veterinary radiologist, who used an identical ultrasound device and a vernier caliper on all rabbits. The bulge was measured immediately after injection of the filler and at 1 day, 3 days, 1 week, and 2, 4, 8, 12, 26, and 38 weeks. The volume of each protrusion was calculated using the formula *V *= height × length × width × 0.5.

### Measurement with Vernier Caliper

First, the experimenter analyzed and observed the bulge, palpated the skin with his finger to determine the edge of the bulge, and then measured the length, width, and height of the bulge with a vernier caliper with an accuracy of 0.01 mm (Shanghai Menite Industrial Co., Ltd., Shanghai, China). The height value is the thickness of the entire elevation at the highest point of the ear minus the thickness of the ear itself at its symmetrical position.

### Measurement with Ultrasonography

Ultrasonic detection was performed using the electronic linear array probe of the MS250 rat Vevo2100 ultrasonic instrument, which has a frequency range of 13–24 MHz. The epidermal layer was identified as having a linear high echo, whereas the raised high-resolution gray image with a uniform and equal echo package was identified as the bulge. The volume of each bulge was calculated using transverse and sagittal views and by measuring the height, width, and length of the area.

### Histological Examination

Rabbits were dissected at 4, 12, 26, and 38 weeks after filler injection, and the implanted sites and surrounding ear tissues were collected and fixed in 12% neutral buffered formalin for paraffin embedding. The tissue was microtomed vertical to the longitudinal axis of the thread at 4-μm thickness. Samples were stained with hematoxylin and eosin and Masson’s trichrome for light microscopy.

### Statistical Analyses

Data are presented as mean ± standard deviation of the mean and later analyzed using the Statistical Package for Social Sciences version 11.5 (SPSS Inc., Chicago, USA). Since the data were not normally distributed, the pre–post comparisons were done using Wilcoxon’s signed-rank test. Differences were considered statistically significant at *P *< 0.05.

## Results

### Observation

#### General Observation

After HA was injected under the skin of rabbit ears, obvious bulging was observed immediately. The skin of rabbit ears showed obvious redness 1–2 weeks after HA injection, with the skin returning to its normal state by week 3. The bulge lasted for 12 weeks, and the outline of the injection area could still be identified 26 weeks after HA injection. At week 38, the contours of some injection areas were not easily distinguishable from those of the surrounding areas. During the observation period, no obvious abnormalities were found in the rabbits.

#### Ultrasound Observation

**Fig. 2:**
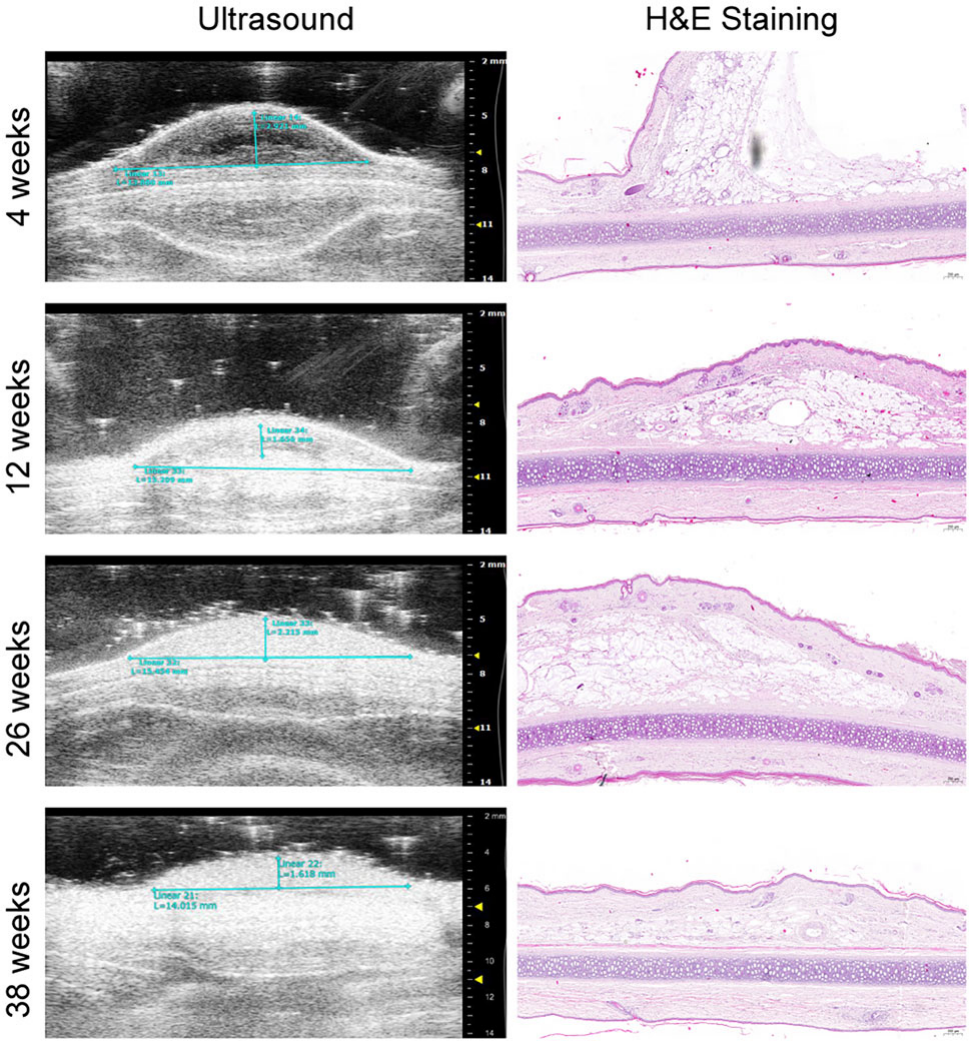
Ultrasound and histopathological results after injection in rabbit ear. Left, ultrasound images at different time points; right, histopathological results under hematoxylin-eosin staining. In the ultrasound images, the injected area can be observed, and the height and width of the region are measured. Hematoxylin–eosin staining reveals that the injected site remains elevated for a long time, and as time progresses, the amount of fibrous tissue gradually increases. Residual material remained visible at 38 weeks

The filler was detected as a well-defined, regular, and hypoechoic mass in the subcutaneous tissue. During examination, it was possible to identify and measure the filler at the injection site. The volume area was determined using the transverse and sagittal views and by measuring the height, width, and length of the area were measured (Fig. 2).

#### Pathological Evaluation

At 4 and 12 weeks after injection, fibrous encapsulation around the products and fibrous tissue ingrowth between the products forming small fibrous capsules were observed. At 26 weeks after injection, fibrous capsules between the products were evident, and polycystic cavities formed as the product degraded. At 38 weeks after injection, product degradation was more obvious than that at 26 weeks, and a very small amount of product could be seen locally (Fig. 2).

### Data Measurement

#### Vernier Caliper Measurement

Compared with that immediately after injection, the height of the bulge increased significantly on the 3th day (2.15 ± 0.46 mm vs. 2.44 ± 0.33 mm, *P *= 0.047) and reached the highest point on the 2nd week. By the 4th week, it remains significantly higher than the immediate post-injection bulge height (2.15 ± 0.46 mm vs. 2.48 ± 0.42 mm, *P *= 0.002), although the height of the bulge decreased significantly on the 8th week (2.15 ± 0.46 mm vs. 1.73 ± 0.58 mm, *P *= 0.035). At 12, 26, and 38 weeks, the height of the bulge further decreased, and at 38 weeks, part of the height of the bulge could not be measured using vernier calipers. The volume significantly increased on the 3rd day after injection compared with that immediately after injection (119.92 ± 27.92 mm^3^ vs. 178.66 ± 35.47 mm^3^, *P *= 0.000). There was no significant difference in the volume of the bulge at week 8 compared with that immediately after injection (119.92 ± 27.92 mm^3^ vs. 178.66 ± 35.47 mm^3^, *P *= 0.112), and the volume of the bulge further decreased (Table 1).

**Table 1:** Vernier caliper measurement of bulge data after HA injection

	Long diameter (mm)	Short diameter (mm)	Height (mm)	Volume (mm^3^)
0	11.43 ± 1.56	9.88 ± 1.20	2.15 ± 0.46	119.92 ± 27.92
1 day	11.64 ± 1.46	10.54 ± 1.26	2.20 ± 0.40	136.28 ± 37.64
3 days	13.06 ± 1.61	11.26 ± 1.36	2.44 ± 0.33	178.66 ± 35.47
7 days	13.79 ± 1.87	11.84 ± 1.05	2.75 ± 0.39	224.75 ± 48.82
2 weeks	13.33 ± 1.52	11.59 ± 0.88	2.97 ± 0.54	228.99 ± 50.44
4 weeks	13.29 ± 1.02	11.33 ± 1.12	2.48 ± 0.42	185.15 ± 32.00
8 weeks	13.28 ± 1.33	11.30 ± 1.32	1.73 ± 0.58	130.60 ± 52.27
12 weeks	13.61 ± 1.36	11.84 ± 1.63	1.43 ± 0.57	118.41 ± 59.05
26 weeks	12.67 ± 2.25	11.33 ± 2.15	0.79 ± 0.45	61.83 ± 41.38
38 weeks	11.81 ± 1.90	10.29 ± 2.17	0.79 ± 0.34	58.92 ± 39.45

#### Ultrasound Measurement

**Fig. 3:**
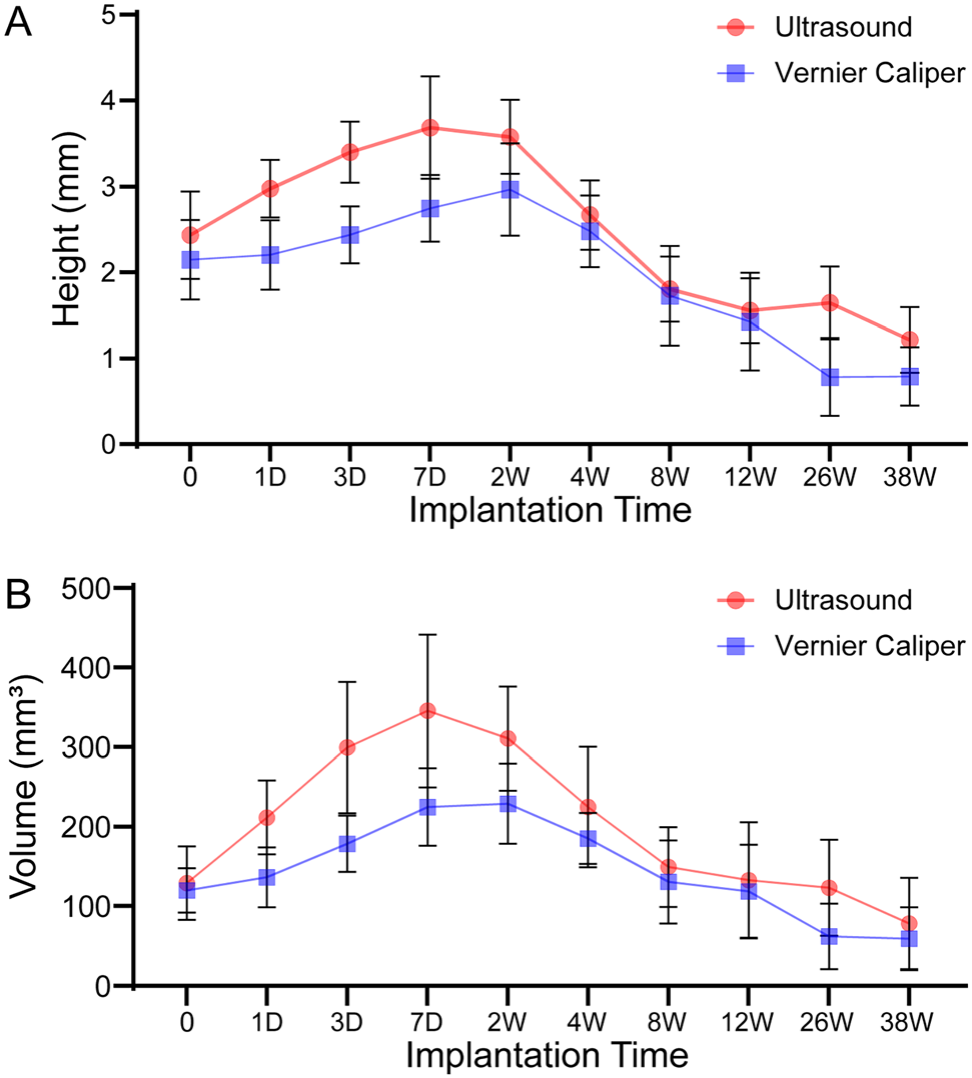
Line chart of the ultrasound and vernier caliper measurement data and calculated data. **A** Measurement data of elevation height; **B** volume calculation data. Both ultrasound and caliper measurements show the same trend

Compared with that immediately after injection, the height of the bulge increased significantly on the 1st day (2.43 ± 0.51 mm vs. 2.98 ± 0.33 mm, *P *= 0.000), but there was no significant difference between the height of the bulge on the 4th week and that immediately after injection (2.43 ± 0.51 mm vs. 2.67 ± 0.40 mm, *P *= 0.058). The height of the bulge decreased significantly on the 8th week (2.43 ± 0.51 mm vs. 1.80 ± 0.38 mm, *P *= 0.000) and further decreased on the 12th, 26th, and 38th weeks. The volume of the bulge increased significantly on the 1st day after injection compared with that immediately after injection (129.09 ± 46.23 mm^3^ vs. 211.63 ± 46.47 mm^3^, *P *= 0.000). There was no significant difference in the volume of the bulge on the 12th week after injection compared with that immediately after injection (129.09 ± 46.23 mm^3^ vs. 149.19 ± 50.29 mm^3^, *P *= 0.647), and the bulge volume further decreased (Table 2). Ultrasonography showed that the height or volume of the colliculus first increased and then decreased, similar to the findings using vernier caliper (Fig. 3).

**Table 2:** Ultrasound measurement of bulge data after HA injection

	Long diameter (mm)	Short diameter (mm)	Height (mm)	Volume (mm^3^)
0	10.71 ± 1.25	9.68 ± 1.29	2.43 ± 0.51	129.09 ± 46.23
1 day	12.72 ± 1.04	11.07 ± 1.02	2.98 ± 0.33	211.63 ± 46.47
3 days	14.21 ± 2.22	12.21 ± 1.25	3.40 ± 0.36	299.27 ± 82.56
7 days	14.66 ± 1.96	12.63 ± 1.47	3.69 ± 0.59	345.44 ± 95.86
2 weeks	14.05 ± 1.67	12.34 ± 1.45	3.58 ± 0.43	310.61 ± 65.34
4 weeks	14.03 ± 1.50	11.74 ± 1.91	2.67 ± 0.40	224.61 ± 75.52
8 weeks	13.65 ± 1.27	11.87 ± 1.74	1.80 ± 0.38	149.19 ± 50.29
12 weeks	13.61 ± 1.97	11.68 ± 2.69	1.56 ± 0.38	132.89 ± 72.69
26 weeks	13.15 ± 1.80	10.96 ± 2.34	1.65 ± 0.42	123.02 ± 60.43
38 weeks	10.87 ± 2.72	10.43 ± 2.94	1.21 ± 0.38	78.23 ± 57.46

## Discussion

As one of the most commonly used body soft tissue fillers, HA not only plays a filling role but also stimulates the production of skin collagen fibers and various growth factors [18–20]. In this study, we demonstrated the efficacy of ultrasound in assessing the filling properties of HA fillers in vivo and found that it could capture uplift data after filling and produce fine-grained data. The presented experimental approach could be a valid and clinically significant method for monitoring the longevity and physical behavior of available tissue fillers, which is important for material engineers when developing and optimizing new dermal fillers and providing the most relevant data for clinical trial studies. Ultrasound is a widely used noninvasive imaging examination with a long history. This can be used as a rapid and accurate method to locate the body filler, observe the tissue around the body filler, control the injection position of the body filler, and simultaneously evaluate the filling degree at the same time [9]. Therefore, ultrasound can be an effective and convenient testing tool for any injected filler and a means of monitoring sediment diffusion, including in animal experiments. The precision of ultrasound measurements is influenced by factors such as the performance of the ultrasound device, the technical expertise of the operator, and the properties of the implanted materials. With a 50 MHz probe, an axial and lateral resolution of less than 100 μm has been achieved. In this study, ultrasound has demonstrated the capability to accurately measure heights smaller than 1 mm, even in the later stages of material degradation, establishing a strong correlation with pathological examinations. Rabbit ear skin is very similar to human skin in certain aspects such as tissue structure and physiological reactions [16, 17]. Therefore, the use of rabbit ear models is a relatively simple and effective method for studying filling effects. The rabbit ear is relatively small and easy to observe visually, providing a clear display of the filling effects and material degradation. This model is important for evaluating the effectiveness and longevity of filling materials and provides valuable data for research and development engineers. In our study, both ultrasound and calipers were used to measure bulging at the injection site, and the differences between them also demonstrate the high precision of ultrasound technology. In contrast to ultrasound, vernier caliper exhibited a certain degree of measurement error when it came to measuring significant or minute bulge. This discrepancy is evident in the context of immediate implant placement, where ultrasound measurements approximate the actual implant volume more closely. It stems from the constrained capacity to accurately delineate the boundaries of the bulge, thus hindering precise measurements. Therefore, ultrasound measurement can obtain finer uplift data after filling and can compensate for the shortcomings of the subjective factors in vernier caliper measurement, which leads to large deviations. The source of HA, degree of crosslinking, and differences in the composition of different products affect the duration of the filling effectiveness of the products [19]. Therefore, an ideal animal model and test can provide quantifiable data and indicators for materials research and development. During in vitro test, materials undergo quantitative testing for bulging degree, rheological properties, cohesion, etc., but these parameters cannot fully reflect the data after implantation in vivo. In our study, histological observations showed that the degree of degradation and tissue-filling ability of HA fillers were consistent with the ultrasound results and were in line with the current maintenance time and degradation cycle of HA fillers in the human body. Ultrasound is a noninvasive detection method that can reduce the number of animals and is more in line with animal ethics. Although nuclear magnetic and fluorescence live imaging are used to evaluate the filling effect and material degradation of fillers, their operation is complex and requires highly technical personnel [21–23]. Therefore, our experimental model can provide improved material parameters for research and development engineers. However, this study has some limitations. In this study, material degradation was not completely observed, and some HA remained in the tissues at 38 weeks. In addition, 3-dimensional graphics were drawn before all images were collected using ultrasonic detection to further quantify the uplift volume. Despite these shortcomings, this experiment proved that the height of the bulge measured by ultrasound after filler injection can be used as an effective index to evaluate the effect of filler injection on animals. There are differences in hyaluronidase between humans and animals, so the degradation of hyaluronic acid will inevitably differ. Although rabbit ears are advantageous for measurement and observation, the tissue structure of the ears differs significantly from the facial anatomical levels of the human body. Our approach initiates with a foundational framework derived from animal experimentation, progressively integrating additional layers of complexity and quantitative variations. This iterative process will inevitably extend to encompass studies involving human subjects. Nonetheless, during the preliminary stages, we aim to establish a robust animal model to initially assess the impartiality and efficacy of our methodology, thus laying a groundwork for future investigations. We will continue to focus on the injection and detection methods for fillers in animals to determine whether the data in animals can fully match clinical applications. Possibly utilizing more comprehensive animal models or clinical trials in humans is needed to assess encapsulation and inflammatory reactions thoroughly. At the same time, we are also considering whether it is possible to determine the optimal interval of supplementary injection for various products through detection using ultrasound and other results in animals.

## Conclusion

Evaluation of the volume impact of dermal filler through the rabbit ear injection model evaluation enables material science evaluation in the early stage of material development, and has certain clinical reference value.
